# Visual perception and color discrimination of the Asian citrus psyllid

**DOI:** 10.1371/journal.pone.0361772

**Published:** 2026-10-30

**Authors:** Sandra A. Allan, Emma N. I. Weeks

**Affiliations:** 1 Insect Behavior and Biocontrol Research Unit, Center for Medical, Agricultural and Veterinary Entomology, Agricultural Research Service, United States Department of Agriculture, Gainesville, Florida, United States of America; 2 Entomology and Nematology Department, University of Florida, Gainesville, Florida, United States of America; University of Saskatchewan College of Agriculture and Bioresources, CANADA

## Abstract

The spectral sensitivity of adult Asian citrus psyllids, *Diaphorina citri* Kuwayama (Hemiptera: Liviidae), a vector of huanglongbing, was determined as a basis for optimization of visual traps. Electroretinogram responses were obtained from compound eyes of dark-adapted psyllids that were exposed to monochromatic 200 ms light flashes ranging from 340–700 nm. The spectral sensitivity curves were comprised of a peak in the ultraviolet region as well as a broad peak in the green-yellow region of the visual spectrum. Curves of male and female psyllids were similar and combined for further analysis. By fitting visual pigment templates to the curves, contributions from photopigments with maxima at 350, 430, 500, and 580 nm were revealed. In paired-choice attraction assays with sticky cards comparing colors at different intensities to grays of matching brightness, yellow and green were strongly attractive across the range of intensities, blue was generally repellent and red was not different than gray in attraction. By aligning specific photopigment sensitivities with behavioral preferences, this study establishes the physiological mechanisms underlying psyllid color attraction to guide the development of targeted visual monitoring tools.

## Introduction

Huanglongbing (HLB) or citrus greening is the most devastating citrus disease globally, causing severe reductions in fruit quality and quantity [1,2]. The Asian citrus psyllid, *Diaphorina citri* Kuwayama (Hemiptera: Liviidae) serves as the primary vector for three *Candidatus* Liberibacter species, of which *Candidatus* Liberibacter asiaticus (CLas) is associated with the decline of the citrus industry in Florida [3]. Given the absence of a cure for the disease, disease containment relies heavily on managing the vector. However effective population control is severely challenged by the development of insecticide resistance [4], high management costs [5], environmental concerns [6], and adverse effects on non-target organisms [7].

Because management relies on monitoring to track infestation levels, tools effective at very low population densities are critical. Understanding the sensory mechanisms guiding *D. citri* host plant location is essential for developing enhanced monitoring and management strategies. While host plant location in many insects involves complex interplay between olfactory and visual cues [8–10], sternorrhychan Hemiptera appear to rely primarily on visual stimuli for initial attraction [11]. Similarly, *D. citri* exhibits strong phototactic behavior, with plant odors functioning primarily as orientation cues in the presence of visual stimuli [12–14]. Adult *D. citri* are highly attracted in both the laboratory and field to yellow and yellow-green sticky traps [15–19] as well as white ultraviolet (UV), green and yellow light emitting diodes (LEDs) [20,21]. Furthermore, studies demonstrated enhanced attraction to yellow or green wavelengths when combined with UV [22,23].

Color perception involves spectral distribution, wavelengths that comprise the color, spectral purity as well as brightness or overall intensity of reflectance [24,25]. The ability to distinguish stimuli based on wavelength composition independent of brightness requires neuronal comparisons of inputs from at least two distinct spectral photoreceptor types [26–28]. While insects typically possess receptors in the UV, blue, and green families, there is considerable variation [29]. The precise spectral sensitivities determine the visual cues driving behavioral choices. Importantly, visual perception does not always equate to behaviorally significant phototrophic responses but could also play a role in motion detection, background contrast or photokinensis and these factors can contribute individually or in combination to the phototrophic response [29].

The objective of this study was to determine the spectral sensitivity of *D. citri* and examine specific wavelengths in landing choices of *D. citri*, which can contribute to the refinement of surveillance and management strategies.

## Materials and methods

**Insects.** Asian citrus psyllids were reared on orange jasmine, *Murraya paniculata* (Linnaeus; Rutaceae) in a climate-controlled greenhouse at 29 ± 3°C under a photoperiod of 16:8 L:D using a combination of natural light and metal halide lamps. Plants were fertilized monthly with Milorganite® (Milorganite, Milwaukee, Wisconsin, USA) and Miracle-Gro® (The Scott’s Company, Marysville, Ohio, USA). Both rearing plants and randomly selected psyllids from the colony were evaluated using qualitative polymerase chain reaction (qPCR) [30] to verify that neither plants nor insects were infected with the *Ca.* L. asiaticus. Host plants were rotated into and out of colony cages at 4–6 week intervals to maintain plant health. Adult *D. citri* obtained from the colony for assays represented a mix of ages and physiological states presumably similar to field populations.

### Electrophysiological recordings

Electroretinograms (ERG) were produced by the flash method [31] to calculate the spectral sensitivity of adult psyllid compound eyes. These extracellular recordings reflect contribution from the multiple individual photopigments present. Adult *D. citri* were immobilized in sticky wax (Cenco, Central Scientific Company, Chicago, Illinois, USA) by gently depressing the wings and legs into soft wax and fastening the head in position with melted wax, taking care not to obstruct the eyes or spiracles. Recordings were conducted in a dark room in a grounded Faraday cage covered with blackout cloth. Electrodes consisted of electrolytically sharpened tungsten wires (0.127 mm, A-M Systems, Carlsburg, Washington, USA) with the indifferent electrode inserted into the thorax. The recording electrode was inserted into the center of the left eye in the center of a light beam delivered from a UV-VIS quartz optic fiber (6.3 mm diam, Dolan-Jenner Industries, Boxborough, Massachusetts, USA) positioned with a micromanipulator 1 cm from the eye surface. If a test stimulus at 560 nm resulted in a typical ERG response, i.e., a standard negative waveform, then the psyllid was dark-adapted for 1 hr.

The light stimulus consisted of monochromatic light produced from a halogen lamp (EXR 300W/82V, OSRAM, Houston, Texas, USA) after it was transmitted through one of a series of fifteen 10-nm narrow bandpass interference filters (Edmund Industrial Optics, Barrington, New Jersey, USA) representing wavelengths between 340 and 700 nm. Quantal stimulation at each wavelength was controlled with neutral density filters to adjust light flux as needed. Filters consisted of a circular variable neutral density filter (Edmond Optics, Barrington, New Jersey, USA) (0.04–4.00 optical density (OD) placed in a 360^o^ wheel mount with stops at every 10^o^ as well as additional neutral density filters ranging from 0.5–4.0 OD (5 × 5 cm, Wratten gelatin filters; Eastman Kodak Company, Rochester, New York, USA) placed in a holder in the light stream. Light stimulus consisted of a pulse of light (200 ms) provided by a manual photographic shutter.

The ERG measurement consisted of the voltage difference between the baseline and the peak of the ERG component. Voltage responses were amplified by a 10X amplifier associated with the electrode (EAG COMBI 10X, Syntech, Hilversum, The Netherlands) and 10X by an additional amplifier (Syntech Autospike IDAC) then captured on a computer using Syntech EAG software (ver. 2.4 Syntech, Kirchzarten, Germany). The optical system was calibrated for irradiance with a concave grating spectroradiometer (Black comet CXR-SR, StellarNet Inc, Tampa, Florida, USA) with a quartz light guide and quantal flux was calculated. Psyllids were exposed to a maximum intensity of 5.2 × 10^15^ photons/cm^2^/sec (520 nm), whereas a typical daylight (sunlight) intensity is 3.6 x10^13^ photons/cm^2^/sec [32].

Preliminary tests at different light intensities at 380, 430, 520, and 580 nm were used to produce intensity-response curves that followed a log-linear function for a portion of the curve. Response voltage versus log of light intensity (uW/cm^2^/s) (V-*log*I) curves of data from male or female *D. citri* were plotted and the criterion response of 3 mV selected. Following dark adaptation, each *D. citri* was exposed to light passed through all 15 filters to produce ERG responses. Measurements using filters below 340 nm were at or below level of detection and not included in the study. Voltage measurements started with low light intensities and light intensities increased until the criterion response was obtained. At the beginning, at intervals throughout the recording and at the end, responses were measured of a low intensity exposure to 560 nm and compared to determine if the preparation had degraded through the recording, in which case data were discarded. Each reading was replicated three times then averaged and data for each sex consisted of 10 replicates.

### Spectral sensitivity

Spectral sensitivity was determined by taking the inverse of the photon count needed to trigger a standard response across 15 wavelengths. To find these values, linear regression was used to plot neutral density against photon flux (10^12^); the reciprocal of the resulting photon count at each wavelength provided the sensitivity [33]. Spectral sensitivity curves were calculated for each sex from the mean of all ten individual *D. citri*. To remove differences in absolute sensitivity between individual insects, individual spectral curves were normalized and then averaged. Photopigment sensitivity is characterized by its peak of absorption or λ_max_ [34,35]. The contribution of individual visual pigments with different λ_max_ to *D. citri* spectral sensitivity curves was investigated by comparing measured sensitivity curves to the individual and mixtures of theoretical absorption spectra of visual pigments (nomograms) with different α-peak maxima (λ_max_).

### Behavioral assays

To differentiate if attraction and landing on a surface occurred in response to color or brightness of stimuli, two-choice assays were conducted using a card of a particular color and brightness compared to a gray card of a matching brightness of reflected light. Colors selected were based on the general range of sensitivity of photoreceptors (blue, green, yellow) and red was included due to previous reports of attraction to red targets [36]. These assays were conducted at 27 ± 1^o^ C in clear-topped white screened cages (60 × 60 × 180 cm) (Megaview Science, Taichung, Taiwan) under metal halide lights (250-watt) providing both visible and ultraviolet (UV) light. Cages were placed in an enclosure surrounded by white posterboard or fabric to reduce visual distraction. For each test 65–70 *D. citri* were mechanically aspirated from colony cages and released in the center floor of the assay cage. Assays were conducted for 4 hr then the cards were collected, any remaining *D. citri* were collected, and all *D. citri* were sexed and counted. For the assays, two cards were placed on the back wall of each cage, equidistant from each side and with the sticky side of each card facing into the cage. The background for all tests was the white backing of the cage. Card position (left or right) noted for each replicate and placement switched in subsequent assays to address potential positional bias. Assays were repeated 16 times.

Cards (10 × 10 cm) were made from acetate sheets first painted with white titanium dioxide acrylic primer and then painted with test paint mixtures. Each card was covered on the painted side with clear double-sided sticky sheet (Alpha Scents, West Linn, Oregon, USA) and wires were used to fasten the cards to the cages. Paints were prepared in four paired series: yellow, green, blue and red. Each color was prepared over a range of brightness of reflected light by diluting the pure colored paint with increasing amounts of white paint so that brightness of the color increased with a higher percentage of white paint. Similarly, black paint was diluted with white paint to produce gray paint with different levels of brightness. Paint mixtures were prepared from artist acrylic paints and included titanium white, ivory black, cadmium yellow medium hue, permanent green light, cobalt blue hue, and cadmium red (Galeria Acrylic, Winsor & Newton, London, UK). Intensities of different colors (blue, green, yellow, red) were paired with respective gray paint mixtures developed to approximate in brightness. Reflectance from the different intensities of colored paints and selected grays are presented in S1 Fig. Brightness levels of paired colored and gray paints were assessed as relative quantum catch as a function of illuminance, reflectance and psyllid spectral sensitivity (adapted from 11,37) and are presented in Fig 1. P = relative quantal catch.


$$\begin{array}{c} \text{P}=\;\int_{\text{3}\text{5}\text{0}}^{\text{7}\text{0}\text{0}}\text{L}(\lambda )R(\mathrm{\backslash lambdatextrm}S(\lambda )\text{d}(\lambda ) \end{array}$$

**Fig 1:**
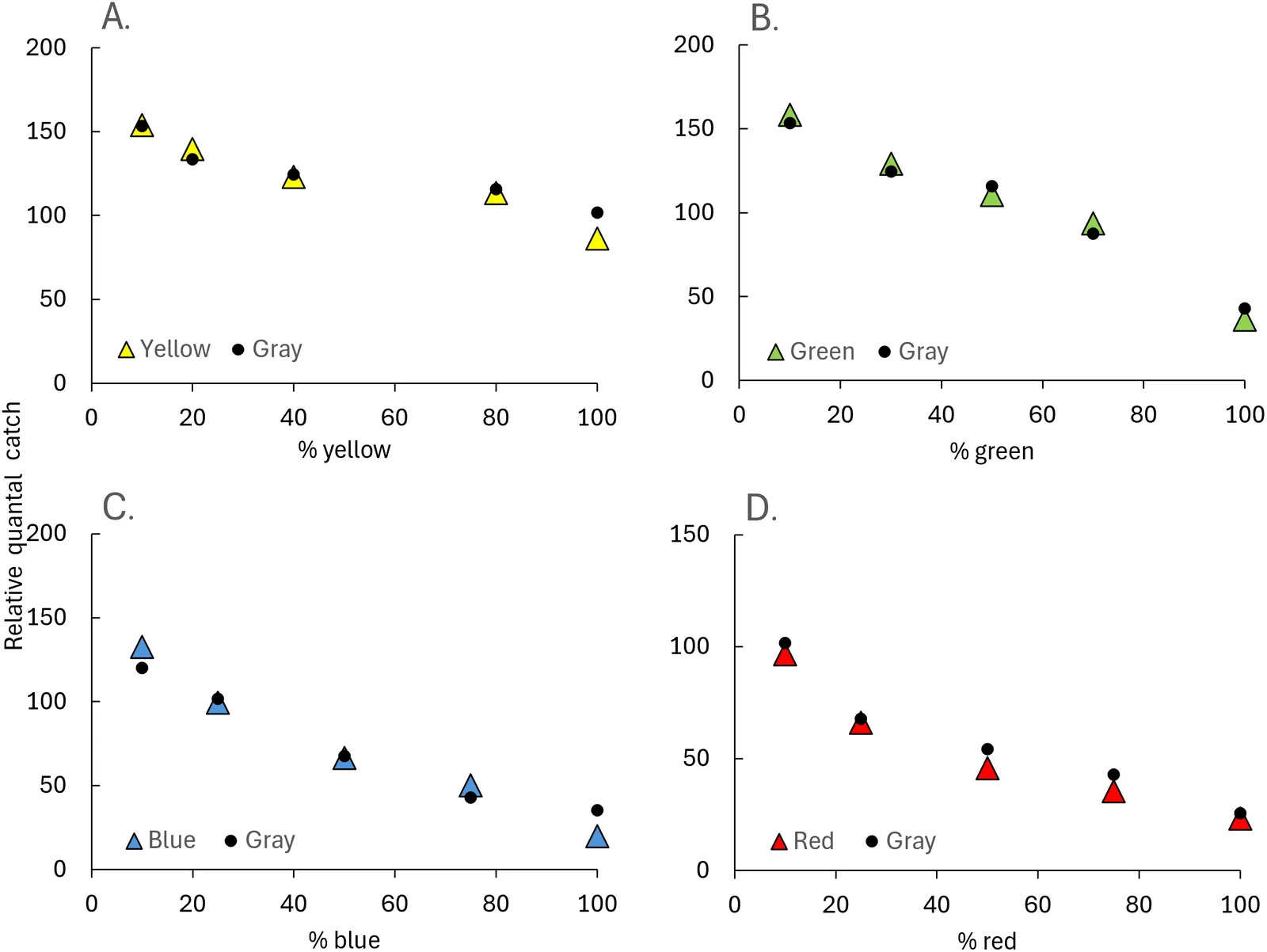
Relative photon catch presented in experiments with paired colors with grays, A. Yellow, B. Green, C. Blue and D. Red. Y axis is % of colored paint mixed with white paint to provide a range of brightness levels. Paired gray paint mixtures were developed to approximate brightness levels of colored paint mixtures. Relative photon catch from each colored or gray paint was based on illumination spectrum, surface reflectance and psyllid spectral sensitivity.

Where L(λ) is irradiance of the illumination source in photons, R(λ) is reflectance of the paint in photons, and S(λ) is the relative sensitivity of the psyllid. All tests were conducted against a highly reflective white background so background compensation was not calculated.

### Reflectance measurements

Cards were illuminated with a deuterium/tungsten/halogen light source (StellarNet, Inc., Tampa, Florida, USA) and a concave grating spectrometer (UV-VIS Black Comet, StellarNet Inc., Tampa, Florida, USA) with quartz light guides was used to obtain spectral reflectance from 300 to 700 nm. Illuminance spectra for the bioassays was similarly measured.

### Statistical analysis

Fit of pigment mixtures to observed spectral sensitivity were analyzed by polynomial regression analysis (SigmaStat, Systat, San Jose, California, USA). Bioassay data were calculated as percentage of *D. citri* collected on cards compared to the number released in the cages (% responders). Of those that responded, the percentage that responded to the color or gray cards also was calculated. Because there was no difference between male and female response to the cards (*t*-test, *P* > 0.05), responses of both sexes were combined. Data normality (Shapiro-Wilk test) and homogeneity of variance (Levene’s test, α = 0.05) were tested (SigmaStat, Systat, San Jose, California, USA). If data were normal, parametric tests (ANOVA, t-test) were used and for non-normal data, non-parametric (Mann-Whitney Rank Sum test) tests were used.

## Results

The typical ERG waveform of a dark-adapted *D. citri* (Fig 2) consisted of a strong monophasic negative on-response followed by a graded return to baseline. The intensity-response curve indicated that the eye responded over at least 4 log units of intensity and the lack of plateauing indicating that responses were not saturated at exposure to the higher levels of light (Fig 2).

**Fig 2:**
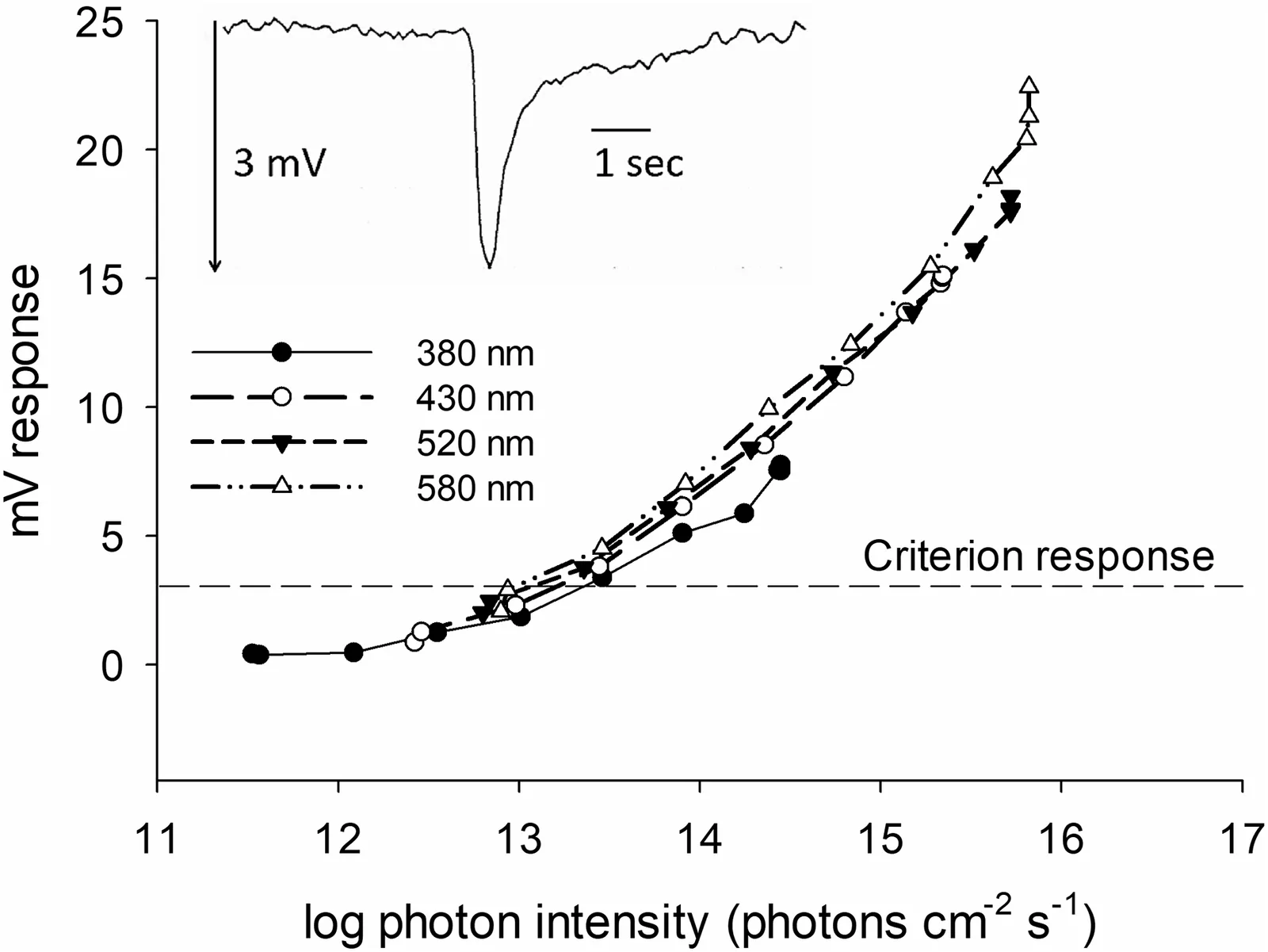
A typical electroretinogram waveform from a dark-adapted *Diaphorina citri* showing depolarization and response vs light intensity (V-logI) curves at 380, 430, 520, and 580 nm. The criterion response of 3 mV is marked by the dashed line.

The spectral sensitivity curves of males and females were characterized by a high responses in the UV, a decrease around 400 nm and a larger broad peak at 500–550 nm with reduced responses above 600 nm (Fig 3). Curves for males and females were very similar with little overall difference in shape and data were combined for subsequent nomogram fitting. Sensitivity curves of individual or combinations of up to three pigments did not account for the observed spectral sensitivity curve (Fig 4, S2 Fig). The best fit of theoretical pigment mixture to the measured ERG values was provided by a 19:21:27:33 ratio of pigments with a λ_max_ of 350, 430, 500, and 570 nm (S1Table).

**Fig 3:**
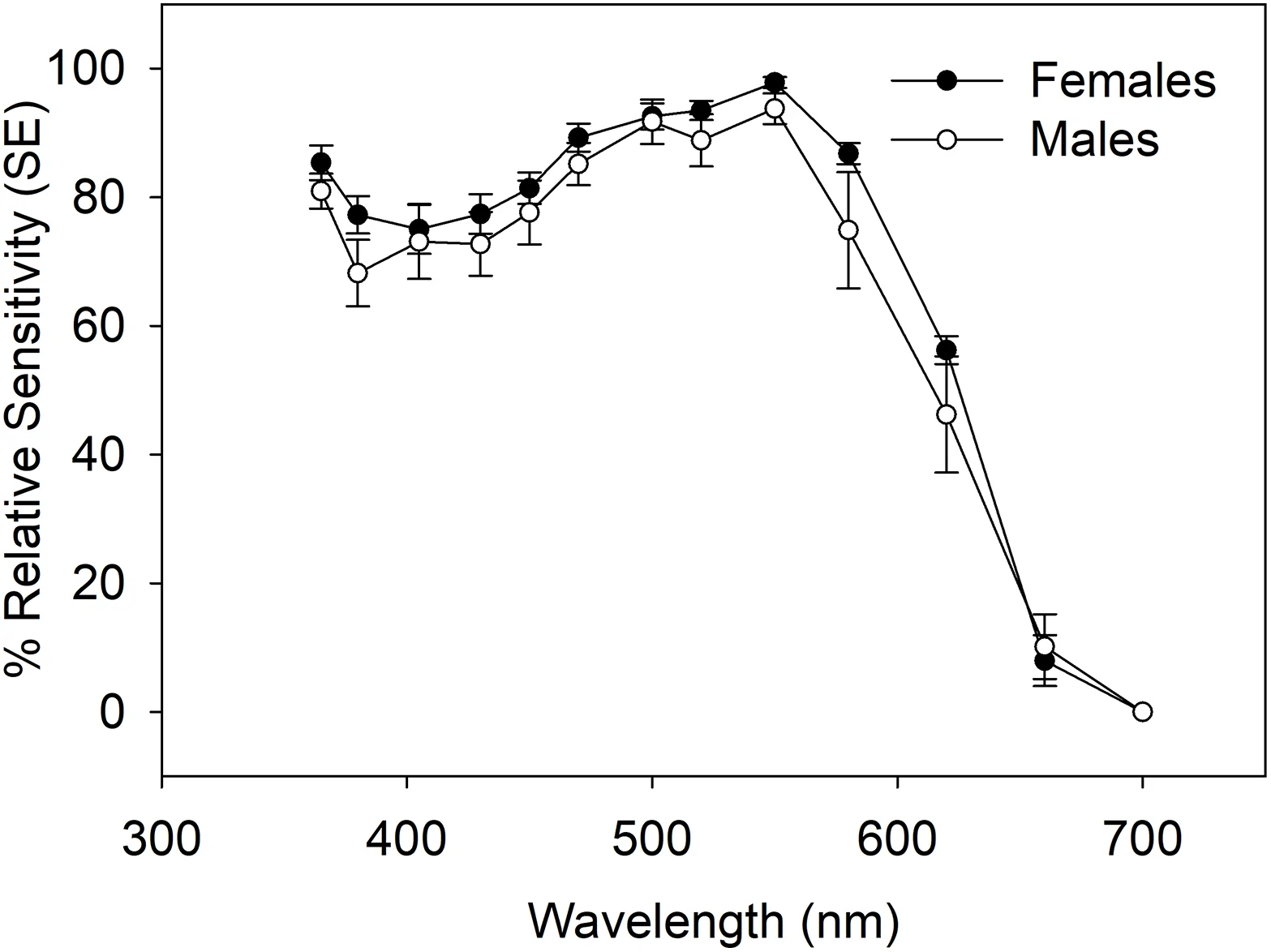
Relative spectral sensitivity curves of male and female *D. citri.*

**Fig 4:**
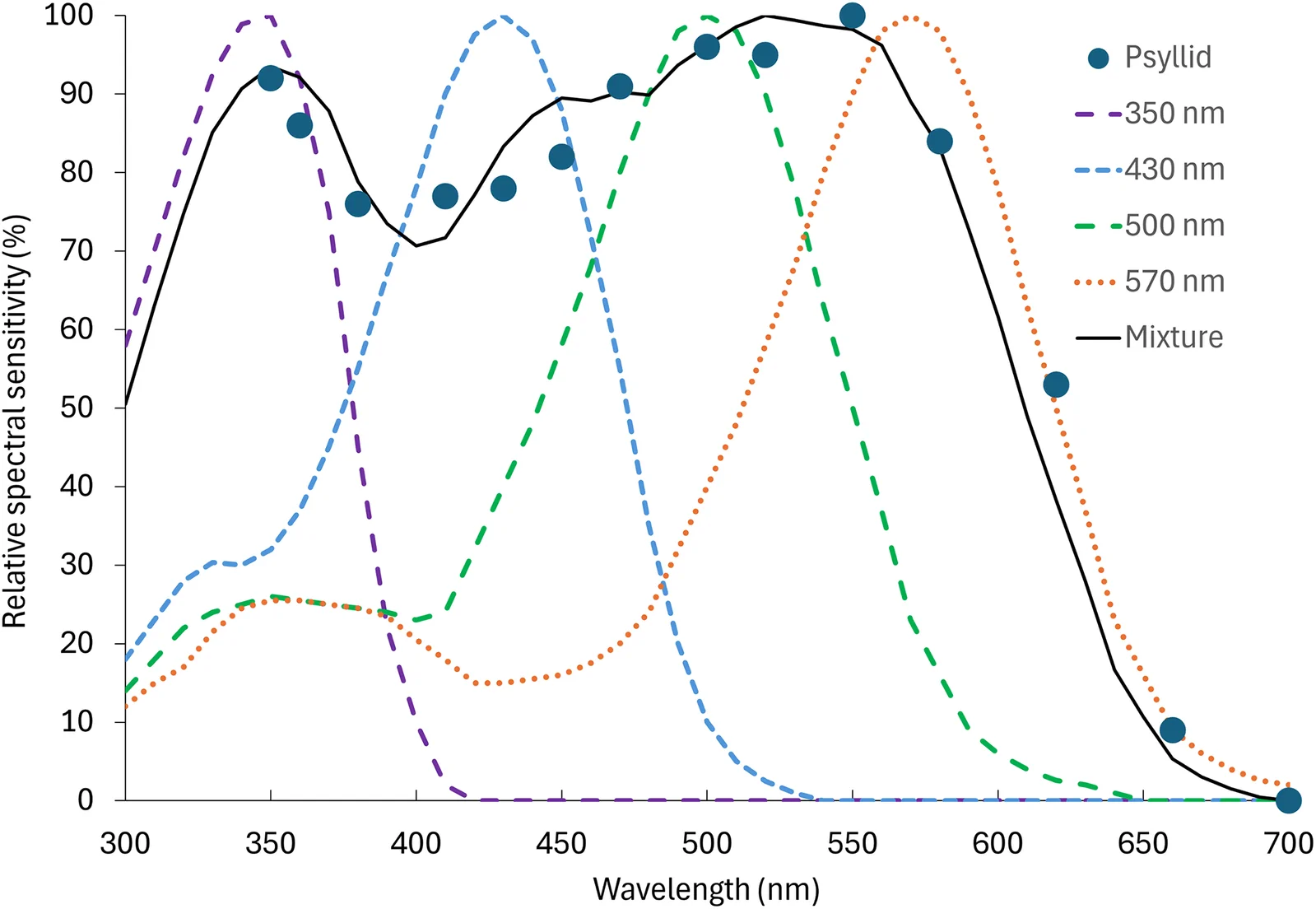
Mean spectral sensitivity values for *D. citri* with individual absorption curves for 350, 430, 500, and 570 nm photopigments. Additionally, a composite pigment mixture consisting of 19% 350 nm, 21% 430 nm, 27% 500 nm, and 33% 570 nm is provided to match the measured psyllid responses.

Color preference was evident during paired trials where the gray cards were presented that matched colored cards in brightness. The percentage of *D. citri* responding in each test varied from 56.48–68.86% for yellow, 54.85–72.89% for green, 29.04–57.16% for blue, and 17.55–27.72% for red (Tables 1–4).

**Table 1:** Responses of adult *Diaphorina citri* to paired sticky cards in two-choice assays in screened cages. One card was coated with a paint mixture of yellow and white, and the other card was coated with a mixture of black and paint to make gray that matched in brightness. All data (males and females combined) were normal and analyzed by paired t-test (α = 0.05). N = 16.

% paint		% responding (SE)	% on cards (SE)		*t*	df	*P*
Yellow	Black		Yellow	Gray			
100%	1.5%	62.62 (3.76)	83.52 (2.62)	16.48 (2.62)	18.01	30	< 0.001
80%	1.0%	67.60 (4.08)	84.65 (2.65)	15.35 (2.65)	18.43	30	< 0.001
40%	0.5%	68.86 (2.67)	83.87 (2.67)	16.13 (2.67)	15.14	30	< 0.001
20%	0.1%	56.48 (3.46)	83.35 (3.09)	16.65 (3.09)	15.22	30	< 0.001
10%	0.075%	59.67 (3.50)	81.17 (3.61)	18.82 (3.61)	12.20	30	< 0.001

**Table 2:** Responses of adult *Diaphorina citri* to paired sticky cards in two-choice assays in screened cages. One card was coated with a green and white paint mixture, and the other card was coated with a mixture of black and white paint to produce gray that matched in brightness. All data (males and females combined) were normal and analyzed by paired t-test. N = 16.

% paint		% responding (SE)	% on cards (SE)		*t*	df	*P*
Green	Black		Green	Gray			
100%	25%	72.89 (2.67)	79.58 (3.78)	20.42 (3.78)	11.06	30	< 0.001
70%	4.0%	59.06 (5.53)	74.07 (4.59)	25.93 (4.59)	7.41	30	< 0.001
50%	0.6%	55.98 (4.92)	79.40 (4.97)	20.59 (4.97)	8.35	30	< 0.001
30%	0.5%	70.51 (11.60)	77.93 (4.10)	22.07 (4.10)	9.62	30	< 0.001
10%	0.2%	54.85 (3.06)	53.06 (3.06)	46.94 (3.06)	0.71	30	0.24

**Table 3:** Responses of adult *Diaphorina citri* to paired sticky cards in two-choice assays in screened cages. One card was coated with a blue and white paint mixture, and the other card was coated with a mixture of black and white paint to produce gray that matched in brightness. Data (males and females combined) that were normal were analyzed by paired *t*-test; those not normal were tested with Mann-Whitney Rank Sum Test (*U*). N = 16.

% paint		% responding (SE)	% on cards (SE)		*U*	*t*	df	*P*
Blue	Black		Blue	Gray				
100%	70%	57.16 (5.87)	27.90 (4.14)	72.09 (4.14)	7.00		30	< 0.001
75%	30%	29.04 (1.79)	44.30 (6.02)	55.70 (6.02)	98.00		30	0.265
50%	15%	35.45 (2.68)	33.97 (4.63)	66.02 (4.63)		4.89	30	< 0.001
25%	0.5%	34.61 (3.40)	48.08 (5.67)	51.92 (5.67)		0.46	30	0.642
10%	0.4%	44.03 (4.53)	40.04 (6.53)	59.95 (6.53)		2.21	30	< 0.05

**Table 4:** Responses of adult *Diaphorina citri* to paired sticky cards in two-choice assays in screened cages. One card was coated with a red and white paint mixture, and the other card was coated with a mixture of black and white paint that produced gray that matched in brightness. All data (males and females combined) were normal and analyzed by paired t-test. N = 16.

% paint		% responding (SE)	% on cards (SE)		*t*	df	*P*
Red	Black		Red	Gray			
100%	50%	26.25 (4.19)	53.65 (6.17)	46.35 (6.17)	0.83	30	0.410
75%	15%	27.72 (5.04)	49.01 (7.12)	50.98 (7.12)	0.19	30	0.846
50%	5%	25.27 (3.91)	54.27 (7.41)	45.73 (7.41)	1.01	30	0.318
25%	2%	17.55 (3.40)	46.11 (8.50)	53.89 (8.50)	0.64	30	0.523
10%	0.5%	16.83 (3.41)	31.60 (8.90)	68.4 (8.90)	2.92	30	0.007

The overall percentage of *D. citri* that were collected on cards (% responders) compared to those released into the cages differed significantly between the colors tested (H = 166.19, df = 3, *P* < 0.001). Responders were highest with yellow (62.94 ± 1.67) and green (59.96 ± 1.89) cards, moderately low with blue cards (39.22 ± 1.88) and lowest with red cards (22.85 ± 1.85). There were no differences in responses between the five levels of intensities of yellow (*H* = 0.0731, df = 4, *P* = 0.999), blue (*H* = 6.43, df = 4, *P* = 0.169), or red (*F* = 1.578, df = 4, 79, *P* = 0.189). However, *D. citri* responses were lowest to the highest brightness of green (10%) compared to the lower brightness. For yellow card collections, there were significantly more *D. citri* collected on yellow cards than any of the gray cards of matching brightness (Table 1). Similarly, more *D. citri* were collected on green cards compared to gray cards of matching brightness at all levels except for the 10% green, which was equal in attraction to the gray card comprising 0.2% black (Table 2). In general, collection on gray cards was either greater than or equal to the blue cards (Table 3). At the lowest (100% blue) middle (50% blue) and highest brightness (10% blue), more psyllids were attracted to the gray cards (Table 3). For red cards, there was no difference in attraction between red and gray cards except for the lowest brightness (10% red) where gray cards were preferred (Table 4).

## Discussion

The spectral sensitivity of *D. citri* follows a typical pattern often seen in insects with a broad peak in the green region of the spectrum as well as a peak in the UV. This curve was best explained by the presence of four photopigments with λ_max_ at 350, 430, 500, and 570 nm. Most insect species studied have green photoreceptors maximally sensitive about 530 nm and UV receptors with a maximum around 350 nm, although there are considerable differences between species in λ_max_ [37,38]. UV photoreceptors are believed to be present in all insect species, although they have not been reported in all species examined [36]. While blue photoreceptors are broadly present in insects, they are absent or appear to be absent in several species [38].

Green photoreceptors, which provide sensitivity to vegetative backgrounds with maximum sensitivity generally ranging from 500–550 nm [38,39] are broadly represented in insects [38,40]. The broad range of spectral sensitivity in our study can potentially be explained by the presence of two photoreceptors in the green domain, one that is of shorter wavelength (500 nm) (green) and a second of longer wavelength (570 nm) (yellow-green). One of the photoreceptor wavelength peaks of *D. citri* was 500 nm which is on the shorter end of the wavelength range for green [38]. However, the pea aphid, *Acyrthosiphon pisum* (Harris) also has a green receptor at the low end of the green range with a maximum at 518 nm [41]. The second longer wavelength at 570 nm represents the upper end of the green photoreceptors. Healthy citrus leaves have a maximum reflectance at 550 nm and new flush tissue has higher reflectance in the yellow-green range [36,42]. HLB-infected citrus can be distinguished from healthy plants by higher reflectance at 560 and 710 nm. Yellowing from the infection alters leaf color to 560 nm (yellow-green), so that infected vegetation has higher overall visible light reflectance compared to uninfected material [43]. Due to the higher wavelength green photoreceptor, this higher reflectance at 560 nm would be detectable. Additionally, strong attraction of *D. citri* to yellow and yellow-green cards may be related to the 560 nm photoreceptor. The presence of two possible photoreceptors within green could allow better sensitivity and discrimination of variations of colors encountered in the green portion of the spectrum. Although *D. citri* discriminates yellow and green from achromatic stimuli of equal brightness, the close proximity of their spectral maxima may limit fine-scale discrimination between the two hues. While positive phototaxis and landing occurs strongly in response to yellow and green targets, photokinesis under longer wavelength light (green, yellow, orange) was no different than to darkness [42]. This was consistent with observations that psyllids in green tree canopies settle and forage. Wang et al. [44] reported that high sensitivity of photoreceptors and presence of both short and long wavelength receptors together could enhance contrast against the background. Similarly, the presence of photoreceptors sensitive in the shorter and longer green wavelengths may facilitate detection by *D. citri* of yellow-green vegetation (new citrus flush, HLB-infected tissue) against the more predominant green leaves.

Ultraviolet photoreception is widespread among insects [38] and *D. citri* specifically exhibit high sensitivity to UV wavelengths. Behavioral studies suggest that UV perception is a fundamental component of host plant finding for *D. citri*. Substrates with high UV reflectance, such as metalized mulch, are known to repel or disorient diverse taxa including thrips and whiteflies, thereby reducing infestation levels [45–47]. Similarly, field studies demonstrate that highly reflective mulches deter *D. citri* colonization, thereby mitigating the transmission of HLB in young citrus groves [48]. While these materials reflect a broad spectrum of wavelengths, the high UV component is considered the primary driver of this avoidance behavior. Conversely, the attenuation or manipulation of UV light also disrupts host-seeking behavior. For instance, UV-blocking canopy films reduce *D. citri* orientation towards host plants [49–51]. Despite its repellence at high intensities, UV emission can serve as an attractant. Laboratory assays demonstrate strong phototaxis toward UV-emitting LEDs, with peak sensitivity at 375 nm [20]. Wavelength specific behavior was exhibited as positive phototaxis under short wavelength (350–405 nm) light but not under longer wavelengths (500–620 nm) with distance walked greatest under UV and blue wavelengths [42]. These responses are considered related to *D. citri* dispersal. Furthermore, the integration of UV with chromatic stimuli is critical. The addition of UV reflectance to yellow or green substrates enhances *D. citri* attraction, while its removal diminishes the response [20]. Additionally, studies showing that incorporating UV-reflective pigments, such as magnesium oxide, into yellow substrates enhanced both trap captures in the field and laboratory probing behavior [19,23].

Consistent with many diurnal insects, *D. citri* possess blue photoreceptors with a peak sensitivity (λ_max_) of 430 nm [38]. Behavioral responses to blue wavelengths appear complex. While often characterized as neutral or eliciting low-level phototactic responses comparable to white light or reflective surfaces [20,21,36,50], blue stimuli have served as effective stimuli in behavioral conditioning assays [14,52]. Field observations remain inconsistent, with yellow sticky traps sometimes outperforming blue ones, while other trials show no significant difference [53]. Our findings indicate that responses to blues were either significantly lower or equivalent to achromatic gray cards suggesting that blue perceived as a distinct color may be repellent or elicit a neutral response based on brightness. Blue sensitivity in *D. citri* also potentially contributes to broader visual processes such as color opponent mechanisms and motion detection [28].

Variations to red stimuli by *D. citri* have been reported. While Kalile et al. [21] and Paris et al. [22] reported moderate attraction to red sticky traps and LEDs, respectively, Kalile et al. [21] found equivalent preference between red and other wavelengths. These responses were possibly due to a similar brightness rather than wavelength. Results from our study indicate that attraction was generally equivalent between red and corresponding gray cards, thus indicating that attraction was based on brightness alone, not color. Results from previous studies may reflect neutral responses to red and a function of brightness, not color.

*Diaphorina citri* exhibits broad spectral sensitivity with peaks corresponding to specific photoreceptor classes, with ERG recordings indicating a specialized visual system tuned to the yellow-green spectrum (520–580 nm) possibly associated with host plant identification. Additionally, sensitivity to both UV and blue portions of the spectrum support visual capabilities based on a trichromatic vision based on UV, blue, and green photoreception. Recordings in this study were obtained from the central portion of the eye and as there can be photoreceptor ommatidial homogeneity across the eye, visual sensitivity may vary according to the presence or grouping of photoreceptors in certain eye regions. Behaviorally *D. citri* can discriminate yellow-green and blue based on chromatic cues rather than achromatic cues as based on innate taxis and landing responses evaluated in this study. This sensitivity relates to the strong behavioral attraction to specific wavelengths including green and yellow, and supports the development of optimized, monochromatic traps to enhance surveillance and improve integrated pest management strategies against citrus greening. *Diaphorina citri* exhibited the highest response levels (54.85–72.89%) in assays where they demonstrated strong preference for a color (green, yellow) and lower response levels (17.55–57.16%) in treatments where the stimuli (red, blue) were neutral or repellent. The findings bridge the gap between behavioral observations and sensory physiology, highlighting the potential of leveraging sensory biases to enhance vector management in the face of insecticide resistance.

## Supporting information

S1 Fig Reflectance curves of colored traps evaluated for comparison of attraction of Asian citrus psyllids against traps of equal brightness gray. A. yellows; B. greens; C. blues; D. reds and E. grays.
(TIF)

S2 Fig Comparison of psyllid spectral sensitivity curve with pigment absorbance curves (characterized by λ_max_). Comparisons consist of one (550 nm), two (40% 470 nm, 60% 550 nm), three (25% 350 nm, 25% 430 nm, 50% 570 nm) and four (19% 350 nm, 21% 430 nm, 27% 500 nm) pigment nomograms (based on Govardovskii et al. 2000). R^2^ values are presented based on fit of pigment curves to the spectral sensitivity curves. (TIF)

S1 Table R^2^ values from regression of different individual and combinations of four pigments compared to the psyllid spectral sensitivity curve. (DOCX)
