# Supplementary material for: Visual perception and color discrimination of the Asian citrus psyllid
Source: PLoS One. 2026 Oct 30;21(10):e0361772. doi: 10.1371/journal.pone.0361772 (PMC13722172; doi:10.1371/journal.pone.0361772)
Supplement: Supplementary file 1 [file pone.0361772.s003.docx]

**S1 Table. R^2^ values from regression of different individual and combinations of four pigments compared to the psyllid spectral sensitivity curve.**

| % composition of pigment mixtures | | | | R^2^ |
| --- | --- | --- | --- | --- |
| 350 nm | 470 nm | 500 nm | 570 nm |  |
| 100 |  |  |  | 0.054 |
|  | 100 |  |  | 0.233 |
|  |  | 100 |  | 0.857 |
|  |  |  | 100 | 0.698 |
| 25 | 25 | 25 | 25 | 0.830 |
| 20 | 20 | 30 | 30 | 0.985 |
| 15 | 15 | 30 | 40 | 0.985 |
| 20 | 20 | 25 | 35 | 0.989 |
| 19 | 21 | 27 | 33 | 0.991 |
